# Draft Genome Sequence of *Arthrobacter gangotriensis* Strain Lz1y^T^, Isolated from a Penguin Rookery Soil Sample Collected in Antarctica, near the Indian Station Dakshin Gangotri

**DOI:** 10.1128/genomeA.00347-13

**Published:** 2013-06-13

**Authors:** Sisinthy Shivaji, Sreenivas Ara, Sunil Bandi, Aditya Singh, Anil Kumar Pinnaka

**Affiliations:** CSIR—Centre for Cellular and Molecular Biology, Hyderabad, Andhra Pradesh, Indiaa; CSIR—Institute of Microbial Technology, Chandigarh, Indiab

## Abstract

We report here the 4.3-Mb genome of *Arthrobacter gangotriensis* strain Lz1y^T^, isolated from a penguin rookery soil sample collected in Antarctica, near the Indian station Dakshin Gangotri.

## GENOME ANNOUNCEMENT

*Arthrobacter gangotriensis* strain Lz1y^T^ is a Gram-positive, pleomorphic, and nonmotile bacterium (1). According to the 16S rRNA gene similarity, the nearest phylogenetic neighbors of *Arthrobacter gangotriensis* strain Lz1y^T^ are *Arthrobacter antarcticus* SPC26^T^ (99.36%), *Arthrobacter sulfureus* DSM 20167^T^ (98.64%), *Arthrobacter psychrophenolicus* AG31^T^ (98.36%), *Arthrobacter kerguelensis* KGN15^T^ (98.14%), and *Arthrobacter cryotolerans* LI3^T^ (97.77%).

The genome of *Arthrobacter gangotriensis* strain Lz1y^T^ was sequenced using Roche 454 (FLX titanium) pyrosequencing platform. The sequencing yielded 86,795,230 bases in 225,283 reads, which is a 20× coverage of the genome. The assembly of the raw data was performed using the GS *de novo* Assembler (version 2.8; F. Hoffmann-La Roche Ltd., Switzerland). Assembly yielded a genome of 4,319,900 bp in 20 contigs. All contigs were ≥2,394 bp, with largest contig of 656,281 bp, and an average contig length of 173,043 bp. All sequences were above the quality score of 40, with a mean quality score of 63.90. The G+C mol% of the genome of *Arthrobacter gangotriensis* strain Lz1y^T^ was found to be 61.40%, which is near the experimentally calculated value of 66% (1). The Prokaryotic Genome Annotation System (PROKKA) pipeline (2) was used for the annotation of the genome; tRNA was predicted by tRNAscan-SE 1.23 (3) and rRNA genes by RNAmmer 1.2 (4). The annotation has 4,082 genes, including 61 tRNAs, 4 rRNAs (5S-16S-23S), and 4,017 protein-coding genes. The annotation has 5 genes for bacterial chemotaxis and 96 genes for membrane transport, including 51 ABC transporters. The annotation also has 32 genes for resistance against antibiotics and toxic compounds, including 4 arsenic resistance genes, 7 mercury resistance genes, 12 copper homeostasis genes, and 3 cobalt-zinc-cadmium resistance genes. There are 4 genes for alkane biosynthesis in the annotation of the genome.

The sequencing of the genome of this bacterium will help in understanding the adaptation of microorganisms under extreme environmental conditions.

### Nucleotide sequence accession numbers.

The draft genome sequence of *Arthrobacter gangotriensis* strain Lz1y^T^ has been deposited in DDBJ/EMBL/GenBank under the accession AOCK00000000. The version described in this paper is the first version, AOCK01000000.
